# Supplementary material for: Dietary non-starch polysaccharides impair immunity to enteric nematode infection
Source: BMC Biol. 2023 Jun 14;21:138. doi: 10.1186/s12915-023-01640-z (PMC10268516; doi:10.1186/s12915-023-01640-z)

**Supplementary Figure 1.**

Worm burdens in mice infected with *Heligmosomoides polygyrus* for 30 days and fed either an AIN93G control diet, or the control diet supplemented with 10% dried chicory leaves. n=6 per group. Shown are means ± S.E.M.

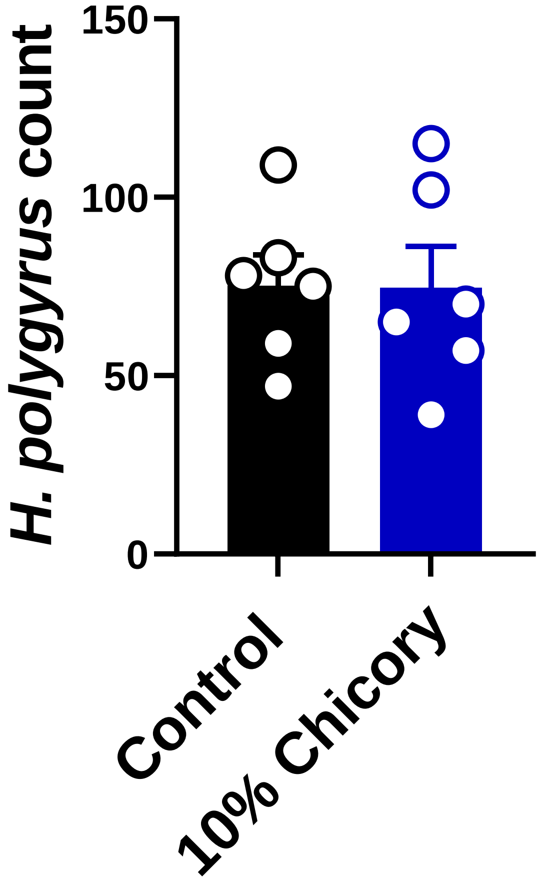

Supplement: Supplementary file 1 — Additional file 1: Supplementary Fig. 1. Effect of dietary chicory during a 30 day Heligmosomoides polygyrus infection. [file 12915_2023_1640_MOESM1_ESM.pdf]
