# Supplementary material for: Dietary non-starch polysaccharides impair immunity to enteric nematode infection
Source: BMC Biol. 2023 Jun 14;21:138. doi: 10.1186/s12915-023-01640-z (PMC10268516; doi:10.1186/s12915-023-01640-z)

Supplementary Figure 2.

Gene expression in the caecum in mice infected for 21 days with 300 *Trichuris muris* eggs, and fed either a control AIN93G diet, the control diet with 5% pectin (and PBS treatment), or the 5% pectin diet (and rIL-25 treatment). Shown are median values. * $p < 0.05$ by Kruskal-Wallis test followed by Dunn's post-hoc testing.

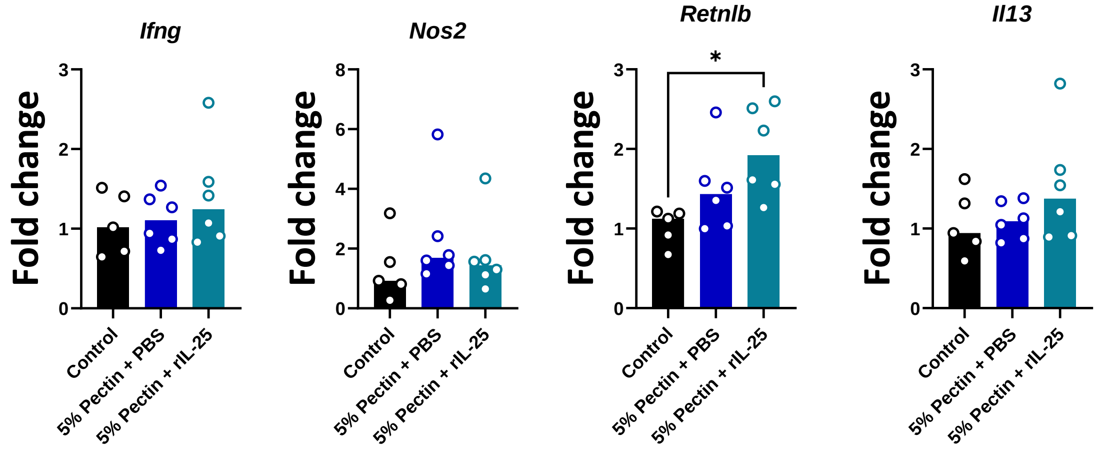

Supplement: Supplementary file 2 — Additional file 2: Supplementary Fig. 2. Caecal gene expression in mice fed pectin with or without IL-25 treatment. [file 12915_2023_1640_MOESM2_ESM.pdf]
