# Supplementary material for: Dietary non-starch polysaccharides impair immunity to enteric nematode infection
Source: BMC Biol. 2023 Jun 14;21:138. doi: 10.1186/s12915-023-01640-z (PMC10268516; doi:10.1186/s12915-023-01640-z)
Supplement: Supplementary file 3 — Additional file 3: Supplementary Table 1. Primer sequences used for qPCR [file 12915_2023_1640_MOESM3_ESM.pdf]

Table S1. Primers used for qPCR

Gene	Primer sequence (5'-3' forward/reverse)
Dclk1	F: ACTGCAGCAGGAGTTTCTGT<br>R: CCGAGTTCAATTCCGGTGGA
Duox2	F: GGACCACGACAGTGATCTCC<br>R: GAACTGTTCCCAGGAGTCCG
Gpx2	F: TCAAACAGTTCACAGGTGGG<br>R: AGTCCTTTAGACCGGTGGGA
Ifng	F: GACTGTGATTGCGGGGTTGTA<br>R: TCACTGCAGCTCTGAATGTTTCT
Il10	F: GCCCTTTGCTATGGTGTCCT<br>R: TAGGGGAACCCTCTGAGCTG
Il13	F: GGCAGCATGGTATGGAGTGT<br>R: CTTGCGGTTACAGAGGCCAT
Nos2	F: GGTGAAGGGACTGAGCTGTT<br>R: TGCACTTCTGCTCCAAATCCA
Retnlb	F: CTGATAGTCCCAGGGAACGC<br>R: GTCTGCCAGAAGACGTGACA
Gapdh	F: TATGTCGTGGAGTCTACTGGT<br>R: GAGTTGTCATATTTCTCGTGG
