# Supplementary material for: Dietary non-starch polysaccharides impair immunity to enteric nematode infection
Source: BMC Biol. 2023 Jun 14;21:138. doi: 10.1186/s12915-023-01640-z (PMC10268516; doi:10.1186/s12915-023-01640-z)
Supplement: Supplementary file 4 — Additional file 4: Supplementary Table 2. Primer sequences used for high-throughput Fluidigm analysis. [file 12915_2023_1640_MOESM4_ESM.pdf]

Primersequences

Gene name	Forward Primer	Reverse primer	Amplicon	Efficiency%
Actb	CCCTAAGGCCAACCGTGAAA	CAGCCTGGATGGCTACGTAC	83	109
Arg1	ATGGGCAACCTGTGTCCTTT	TCTACGTCTCGCAAGCCAAT	127	98
Ccl2	CAGCTCTCTCTTCCTCCACC	TGGGATCATCTTGCTGGTGA	155	110
Ccl3	ACCATGACACTCTGCAACCA	CAACGATGAATTGGCGTGGA	106	103
Ccr10	AAACCCTTGTAGCCAGAGATGG	CGTACCCGGAGTAAAGTCCC	70	104
Cd38	ACTGGAGAGCCTACCACGAA	AGTGGGGCGTAGTCTTCTCT	179	104
Cd3e	CCTCCTAGCTGTTGGCACTT	GGGCACGTCAACTCTACACT	90	107
Cd8a	GGATTGGACTTCGCCTGTGA	TGGGACATTTGCAAACACGC	130	94
Cdh1	GAGACCAGTTTCCTCGTCCG	AGCAGCTCTGGGTTGGATTC	137	105
Cgn	TGAATCCGGGAGCACTGATCT	ACGAAGGGTGCCCATTTCAG	151	106
Cldn1	TCGACTCCTTGCTGAATCTGA	CAGCCATCCACATCTTCTGC	159	109
Cldn7	GCCTTGGTAGCATGTTCCTG	TTTGCTTTCACTGCCTGGAC	180	110
Csf2	ATGCCTGTCACGTTGAATGA	CCGTAGACCCTGCTCGAATA	108	85
Ctla4	ATGGCTTGTCTTGGACTCCG	ACCACTGAAGGTTGGGTCAC	137	103
Ctnnb1	GAGCACATCAGGACACCCAA	CCGAGCAAGGATGTGGAGAG	122	108
Cxcl1	TGCACCCAAACCGAAGTCAT	CTCCGTTACTTGGGGACACC	122	105
Cxcl16	CCCAGATACCGCAGGGTACTT	TTCCCATGACCAGTTCCACA	181	109
Cxcl10	AAGTGCTGCCGTCATTTTCT	CCTATGGCCCTCATTCTCAC	129	101
Cxcr6	TGGAACAAAGCTACTGGGCT	TCGTAGTGCCCATCGTACAG	81	92
Defa3	AAAACTGAGGAGCAGCCAGG	CAGCGACAGCAGAGTGTGTA	194	96
Fasl	AAGGAACTGGCAGAACTCCG	ACTCCAGAGATCAGAGCGGT	151	90
Ffar1	CTGGGCATCAACATACCCGT	AGCAGAAGGCAGTGATGACC	133	92
Ffar4	TGCCCCTCTGCATCTTGTTC	GGTTGGGCCAATCCAATGTG	90	106
Foxp3	AGAGAGAAGTGGTGCAGTCTC	GAGTACTGGTGGCTACGATG	159	97
Fxr	GCTGAGACTGGGTACCAGGG	TCGGAAGAAACCTTTGCAGCC	183	105
Gata3	GCTACGGTGCAGAGGTATCC	CAGAGATCCGTGCAGCAGAG	75	110
Gcg	TCTACACCTGTTCGCAGCTC	GTCCTCATGCGCTTCTGTCT	172	103
Hcar2	CTTCCTACCCAGTGTGGCTG	CAGGTCCACCGAGGAGTAGA	94	106
Hprt	TCAGTCAACGGGGGACATAAA	GGGGCTGTACTGCTTAACCAG	122	86
Icam1	CTGTGCTTTGAGAACTGTGGC	CAGGGTGAGGTCCTTGCCTA	129	101
Ifng	TTTGAGGTCAACAACCCACAG	GCTTCCTGAGGCTGGATTC	94	96
Il10	AGGCGCTGTCATCGATTTCT	ATGGCCTTGTAGACACCTTGG	104	108
Il12b	TTGTTCGAATCCAGCGCAAG	TTCTCTACGAGGAACGCACC	83	105
Il15	ACAGCTCAGAGAGAATCCACC	ATGAGCTGGCTATGGCGATG	187	89
Il17a	TGAGTCCAGGGAGAGCTTCA	CGCTGCTGCCTTCACTGTA	80	89
Il1b	GCAACTGTTCCTGAACTCAACT	ATCTTTTGGGGTCCGTCAACT	89	100
Il2	GAAACTCCCCAGGATGCTCA	CGCAGAGGTCCAAGTTCATCT	99	88
Il27	GTCCACAGCTTTGCTGAATCT	CGAAGTGTGGTAGCGAGGAA	149	99
Il33	GGGCTCACTGCAGGAAAGTA	TTTGCCGGGGAAATCTTGGA	115	101
Il4	CCTGGATTCATCGATAAGCTG	TCCATTTGCATGATGCTCTT	93	99
Il6	GACAAAGCCAGAGTCCTTCAGA	AGGAGAGCATTGGAAATTGGGG	113	102
Irf3	CCACAAGGACAAGGACGGAG	CCACATTTCCCCCATGCAGA	124	101
Itgax	GAGCCAGAACTTCCCAACTG	ACCCGAGCCATCAATCAGG	79	96
Muc1	AGTACCAAGCGTAGCCCCTA	GTGGGGTGACTTGCTCCTAC	118	108
Muc2	TATGCCAGGCCAGGAGTTTA	GCAAGGCAGGTCTTTACACA	82	101
Muc4	GTCCACTTCTTCCCCATCTCG	CCATTGTGACAGTAGCCCTCA	173	103
MVP1	GGAGCCCAGTGTAGAAGAGCA	AGCCAGCGAACCATATCCTGA	87	-
Myd88	CCAGGTGTCCAACAGAAGC	CTTGGTGCAAGGGTTGGTAT	114	101
Nfkb	GGCAGGTATTTGACATACTAAATGG	TGCAGAGTTGTAGCCTCGTG	117	102
Nfkbia	GAGCGAGGATGAGGAGAGCTA	GGCCTCCAAACACACAGTCA	83	107
Nod2	TGGCCCTACAGCTGGATTAC	TTGTTGTTGAAGAGACTGGCTA	187	110
Ocld	GCTGCTGCTGATGAATATAATAGACT	TCCCACCATCCTCTTGATGT	120	104
Pgk1	GGTGTTGCCAAAATGTCGCT	GGACTTGGCTCCATTGTCCA	183	109
Pla2g2a	GGGGCCAAATCACCTGTTCT	GTTCCGGGCGAAACATTCAG	92	105
Ppara	AACATCGAGTGTCGAATATGTGG	CCGAATAGTTCGCCGAAAGAA	99	103
Pparg	TTCAGAAGTGCCTTGCTGTG	CCAACAGCTTCTCCTTCTCG	84	105
Ppia	CCACCGTGTTCTTCGACATC	AGTGCTCAGAGCTCGAAAGT	113	112
Prkaa2	GCAAAGTGAAGACTACCAGGTG	GTAATCCACGGCAGACAGGA	163	110
Pyy	GCTTCTCCCACCTTCCATCT	AGACAGGCGAGCAGGATTAG	121	106
Reg3g_v2	CCCTCAGGACATCTTGTGTCT	ACCTCTGTTGGGTTCATAGCC	141	103
Reg3g	ACAGACAAGATGCTTCCCCG	AGCTGCTACGTGAAGATGGG	127	105
Retnlb	CTGTCCTGCTGGGATGGT	CCAGTCCATGACTGAGCACT	109	98
Sdha	ATTGCTACTGGGGGCTACGG	GTCCTGGCAAGGCAAACCAG	108	106
Stat4	GAAGTACCTCTACCCTGACATTCC	AGGGGACGTAACCCTTGTCT	113	99
Stat5	GGTCCCTGAGTTCGTCAATG	GGTTGGGTGGGTACATGTTG	116	110
Tbp	ACCTTATGCTCAGGGCTTGG	TGCCGTAAGGCATCATTGGA	83	88
Tbx21	GGGCTTCCAACAATGTGACC	AGCTGAGTGATCTCTGCGTTC	193	105
Tff3	CTGTCACATCGGAGCAGTGT	CAGGGCACATTTGGGATACT	67	107
Timp1	GGGGTGTGCACAGTGTTTC	GACCTGATCCGTCCACAAAC	81	107
Tlr2	GCATCCGAATTGCATCACCG	ACAGCGTTTGCTGAAGAGGA	136	99
Tlr3	GAATCACAATCGCGCACCAA	CCATAGGGACAAAAGTCCCCC	178	85
Tlr4	CTCTCATGGCCTCCACTGGT	TTAGGAACTACCTCTATGCAGGGAT	137	104
Tlr5	GATGGATGCTGAGTTCCCCC	AAAGGCTATCCTGCCGTCTG	139	91
Tnfa	CAAATGGCCTCCCTCTCATCA	TGGGCTACAGGCTTGTCAC	88	110
Tnfsf15	CCATCCTCGCAGGACTTAGC	TGCCTCTGGGAGGTGAGTAA	135	101
Tuba	TGTCCTGGACAGGATTCGC	CTCCATCAGCAGGGAGGTG	115	108
Ywhaz	GAAAAGTTCTTGATCCCCAATGC	TGTGACTGGTCCACAATTCCTT	134	111
Zbtb16	GCACTACAGGGTTCACACAGG	CACCGTTGTGTGTTCTCAGG	107	104
